# Comparison of ^99m^Tc-N-DBODC5 and ^99m^Tc-MIBI of Myocardial Perfusion Imaging for Diagnosis of Coronary Artery Disease

**DOI:** 10.1155/2013/145427

**Published:** 2013-06-11

**Authors:** Haiyan Ma, Sijin Li, Zhifang Wu, Jianzhong Liu, Haiyan Liu, Xiaoshan Guo

**Affiliations:** Department of Nuclear Medicine, First Affiliated Hospital of Shanxi Medical University, 85 Jiefang South Road, Taiyuan, Shanxi 030001, China

## Abstract

Despite recent advances in therapeutic and diagnostic approaches, coronary artery disease (CAD) and its related cardiac disorders represent the most common cause of death in the United States. Nuclear myocardial perfusion imaging (MPI) technologies play a pivotal role in the diagnosis and treatment design for CAD. Recently, in order to develop improved MPI agents for diagnosis of CAD, ^99m^Tc-[bis(dimethoxypropylphosphinoethyl)-ethoxyethyl-amine(PNP5)]-[bis(N-ethoxyethyl)dithiocarbamato(DBODC)]nitride(N-DBODC5)(^99m^Tc-N-DBODC5) with a faster liver clearance than conventional single-photon emission computed tomography (SPECT) imaging agents (technetium 99m sestamibi (^99m^Tc-MIBI) or technetium 99m tetrofosmin) has been introduced. In preclinical and phase I studies, ^99m^Tc-N-DBODC5 has shown characteristics of an essentially ideal MPI tracer. Importantly, however, there is no data to support the use of ^99m^Tc-N-DBODC5 to evaluate myocardial ischemia in patients with suspected CAD. The present study was designed to assess the clinical value of this agent; the findings of stress and rest MPI after the administration of this agent were compared to those of stress and rest ^99m^Tc-MIBI, as well as those of coronary angiography, with respect to the detection of CAD. Our findings indicated the usefulness of ^99m^Tc-N-DBODC5 as a promising MPI agent.

## 1. Introduction

Coronary artery disease (CAD) remains the single greatest cause of death in men and women in the USA, despite a declining total death rate. Using 2005 data, over 445,000 (or 1 in every 5) deaths in the USA were due to CAD, and it ranked highest among all disease categories in hospital discharges [1]. CAD also remains the third leading cause of death of the Chinese population, creating a significant socioeconomic burden [2]. Therefore, the reduction of the morbidity and mortality due to CAD is of primary importance to physicians and patients. 

Nuclear cardiology, cardiovascular magnetic resonance, cardiac computed tomography, position emission computed tomography, and coronary angiography (CA) are imaging modalities that have been used to measure myocardial perfusion, left ventricular function, and coronary anatomy for clinical management and research [3]. Based on current guidelines, invasive CA is a suitable diagnostic procedure for patients with a high pretest likelihood of significant coronary artery disease either with or without troublesome symptoms or clinical findings [4, 5]. In this population, the reported diagnostic yield of invasive CA is 44–48% [6–8]. However, in reality, invasive CA has an even lower diagnostic yield of obstructive CAD of approximately 38% [9]. Noninvasive testing could be of value to defer from invasive diagnostic procedures. Stress myocardial perfusion imaging (MPI) has emerged as an important noninvasive mean of evaluating patients with suspected CAD, with over 8.5 million evaluations performed annually in the USA [10].

The most commonly used imaging modality for this purpose is single-photon emission computed tomography (SPECT) [11]. The advantages of nuclear MPI for the detection of CAD are as follows: first, it enables the simple, safe, and noninvasive assessment of myocardial ischemia and reduction of coronary flow reserve using exercise or pharmacological stress. In addition, it provides left ventricular functional information on coronary arteries, which is different from the morphological information provided by CA [12]. Furthermore, CA is the standard technique for assessing epicardial coronary anatomy, and MPI is the standard technique for assessing myocardial perfusion and is appropriate for the quantitative evaluation of myocardial perfusion conditions [13]. 

Thallium-201 (^201^Tl), technetium 99m sestamibi (^99m^Tc-MIBI), and technetium 99m tetrofosmin (^99m^Tc-tetrofosmin) are three traditional, routinely used, MPI tracers and are clinical-validated tracers for evaluation of SPECT MPI [14–16]. Because ^201^Tl is a cyclotron-produced isotope, it is expensive, and it is not easily available for routine clinical use in many developing countries. Therefore, from a practical standpoint, ^99m^Tc-MIBI or ^99m^Tc-tetrofosmin is the preferred agent for a gated SPECT study. However, tracer activity below the diaphragm is commonly seen with ^99m^Tc-MIBI and ^99m^Tc-tetrofosmin, and this can reduce accuracy in some studies [17, 18].

Recently, ^99m^Tc-N-DBODC5 with a faster liver clearance than conventional SPECT imaging agents has been introduced. It is a new lipophilic, monocationic ^99m^Tc-labeled compound that is currently under clinical investigation as a MPI agent [19–24]. Basic research and phase I studies have shown the safety, excellent biodistribution, and high image quality of this radiopharmaceutical [22, 25, 26]. Like ^99m^Tc-MIBI and ^99m^Tc-tetrofosmin, ^99m^Tc-N-DBODC5, also a monocationic lipophilic complex, is possibly localized in the mitochondrial fraction. Marmion et al. [27] and Bolzati et al. [28] reported that the lipophilic characteristics and electronic charge of the tracer were thought to be important. Importantly, however, the clinical value of diagnosis of CAD has not been fully explored.

In order to evaluate the clinical value of this agent, the findings of stress and rest MPI after the administration of this agent were compared to those of stress and rest ^99m^Tc-MIBI MPI, as well as those of CA, with respect to the detection of CAD.

## 2. Methods

### 2.1. Patient Population

Of 120 patients admitted to the hospital because of chest pain from March 2010 to December 2011, 46 (31 males; mean age, 60.08 ± 8.58 years (Table 1)) who underwent stress-rest ^99m^Tc-N-DBODC5 SPECT, ^99m^Tc-MIBI SPECT, and coronary arteriography were included in this study. If the creatine kinase concentration was more than twice the upper normal limit or there was positive troponin T, or if there was evidence of previous myocardial infarction, coronary angioplasty, or a coronary artery bypass graft surgery, the patients were excluded.

### 2.2. Preparation of ^99m^Tc-N-DBODC5

^99m^Tc-N-DBODC5 was prepared using a lyophilized kit formulation purchased from Beijing Shihong Pharmaceutical Center. The saline solution of sodium pertechnetate (1.0 mL, 1110 MBq) was added to an SDH vial (Vail A: 5.0 mg of succinate dehydrogenase, 5.0 mg of 1,2-diaminopropane-N,N,N′,N′-tetraacetic acid, and 0.05 mg of SnCl_2_·2H_2_O). Then, the solution was kept at room temperature for 15 min to form a [^99m^TcN]^2+^ intermediate. The other two lyophilized vials (Vail B: 2.0 mg of PNP5, Vail C: 2.0 mg of DBODC) were dissolved with 1.0 mL of saline solution, respectively. And then the saline solutions of Vails B and C were added to the vial of [^99m^TcN]^2+^ intermediate (Vail A). The resulting solution was heated at 100°C for 15 min. Before injection, the solution was filtrated through a 0.22-*μ*m membrane. The ^99m^Tc-MIBI was obtained using a lyophilized kit formulation (Beijing Shihong Pharmaceutical Center) in the First Affiliated Hospital of Shanxi Medical University.

Radiochemical purity (RCP) was determined by thin-layer chromatography (TLC). TLC was conducted on polyamide film as the station phase and saline/acetone (v / v = 6 : 1) as the mobile phase. Retention factor (Rf) for ^99m^Tc-N-DBODC5 is 0.3–0.6. The RCP was more than 95% in each experimental study, and the purity was still greater than 95% at least 6 h. 

### 2.3. Exercise Protocol

**Figure 1:**
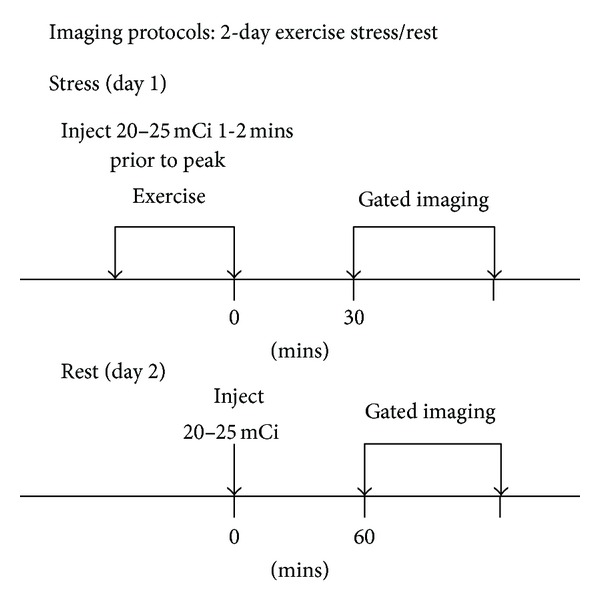
^
99m^Tc-N-DBODC5 and ^99m^Tc-MIBI imaging protocols: two-day exercise stress/rest.

Figure 1 shows that a two-day protocol was used for both ^99m^Tc-MIBI and ^99m^Tc-N-DBODC5 MPI. Image acquisition procedures conformed to the ASNC imaging guidelines [29, 30], and the aged-adjusted maximal heart rate was the endpoint on an ergometer, but other endpoints included physical exhaustion, uncommon arrhythmia, severe angina, or significant hypotension. At the endpoint of the symptom-limited bicycle ergometer exercise, 740 MBq of ^99m^Tc-N-DBODC5 (mean injected activity, 740.8 ± 11.1 MBq) was injected; exercise was continued for a further 2 min. 

Each patient underwent an exercise ^99m^Tc-MIBI study within 7 days of the ^99m^Tc-N-DBODC5 study. For the ^99m^Tc-MIBI (741.5 ± 12.3 MBq) SPECT study, the exercise protocol of patients was identical to that for the ^99m^Tc-N-DBODC5 study. ^99m^Tc-N-DBODC5 and ^99m^Tc-MIBI were used as myocardial perfusion agents in a random sequence. 

### 2.4. Gated SPECT Acquisition and Image Reconstruction

Patients were imaged in the supine position with their arms raised. SPECT images were acquired with a fixed 90° two-headed gamma camera (Infinia VC Hawkeye, General Electric, USA), using a low-energy, high-resolution, parallel-hole collimator, from the 45° right anterior oblique to the 45° left posterior position. Acquisition parameters were as follows: detectors at 90°, 180° rotation at 3° steps with automatic body contouring, 35 s acquisition per step, 64 × 64 matrix, zoom ×1.28, and energy window 140 keV ± 10%. The total acquisition time was approximately 20 min. The same filters were used for both tracers. The raw projection datasets were filtered with a Butterworth filter (cut off frequency 0.50 cycles/pixel and power 6.0 for rest images, cut off frequency 0.50 cycles/pixel and power 5.0 for stress images). No scatter or attenuation correction was performed.

### 2.5. Heart-to-Organ Analysis

^99m^Tc-N-DBODC5 and ^99m^Tc-MIBI heart-to-organ count ratios were calculated from the anterior projection of each tomographic acquisition. Regions of interests (ROIs) were drawn around the entire left ventricular myocardium, over the hepatic margin adjacent to the inferoapical wall of the left ventricle, excluding the biliary tree, and over the inferior left ventricular wall adjacent large intestine activity. The mean counts per pixel in the three ROIs were normalized to the injected tracer activity after decay correction and to a standard acquisition time of 1 min. Heart-to-liver and heart-to-intestine ratios were then computed [31]. 

### 2.6. Image Quality Assessing

For MPI analyses, at first, two experienced observers judged which image sets from ^99m^Tc-MIBI and ^99m^Tc-N-DBODC5 were superior in image quality on the basis of patient motion, statistical noise, tracer activity below the diaphragm, heart-to-organ count ratio, and sharpness, without knowledge of the radiopharmaceutical or patient identity [32].

### 2.7. MPI Image Interpretation

Both overall qualitative diagnosis and semiquantitative 17 segment with 5-point [33] (0 = normal, 4 = absent tracer uptake) scorings were employed in the independent, blinded read by two expert readers who were experienced in SPECT MPI interpretation. In this model, the left anterior descending artery (LAD) distribution territory comprises seven segments (segments 1-2, 7-8, 13-14, and 17), the left circumflex artery (LCX) comprises five segments (segments 3-4, 9-10, and 15), and the right coronary artery (RCA) comprises five segments (segments 5-6, 11-12, and 16). SPECT stress/rest studies were classified for each myocardial region in the following manner: normal if all the segments were normal after stress; ischemic if at least one segments improved at rest; and scar if no segments improved at rest. In addition, the summed stress (SSS), summed rest (SRS), and summed difference scores (SDSs) were calculated, and ischemia was defined as SDS ≥ 2 [34]. The ^99m^Tc-MIBI and ^99m^Tc-N-DBODC5 images were separately interpreted without knowing the clinical histories, results of coronary angiography, or other radionuclide findings. 

### 2.8. Coronary Angiography Interpretation

All coronary angiograms were interpreted with quantitative CA in a coronary angiography core laboratory blinded to the clinical or imaging results. A coronary stenosis was considered present when there was a stenosis ≥ 50% in diameter in any epicardial coronary artery. The presence of one or more coronary stenoses defined the presence of significant CAD.

### 2.9. Statistical Analysis

All statistical analyses were performed using the statistical package SPSS 16.0 (Chicago, IL, USA). The results were expressed as the mean value ± SD. The difference in left ventricular function parameters and perfusion scores were compared using a paired Student's *t*-test. Comparison of the proportion was made with the McNemar test. CA results were used as the “gold standard.” Agreements between CA and SPECT results were defined as the kappa (*κ*) value. *P* < 0.05 was considered significant.

## 3. Results

### 3.1. Coronary Angiography

Of the 46 patients studied, 29 had ≥50% luminal diameter stenosis in at least one major coronary vessel. Fourteen had single-vessel disease, 11 had two-vessel disease, and four had three-vessel disease; 15 patients showed significant stenosis in the RCA, 19 in the LAD, and 14 in the LCX. The remaining 17 patients had normal or nonsignificantly stenosed coronary arteries.

### 3.2. Heart-to-Organ Count Ratio

**Figure 2:**
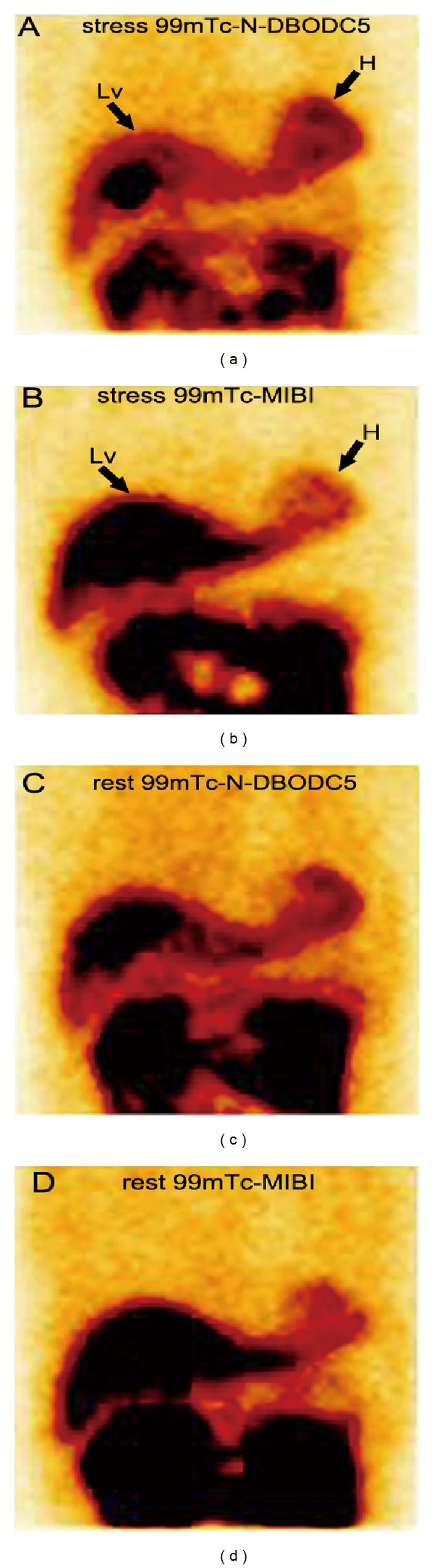
Comparison of liver clearance of the two tracers in anterior tomographic planar images of a patient (as shown in the black arrow; H, heart; Lv, liver). (a) Exercise stress ^99m^Tc-N-DBODC5, (b) Exercise stress ^99m^Tc-MIBI, (c) rest ^99m^Tc-N-DBODC5 and (d) rest ^99m^Tc-MIBI.

Qualitative analyses of images acquired of ^99m^Tc-N-DBODC5 both at rest and during stress revealed no significant overlap between tracer accumulation in the inferior wall of the ventricle and in the subdiaphragmatic region (Figure 2).

**Figure 3:**
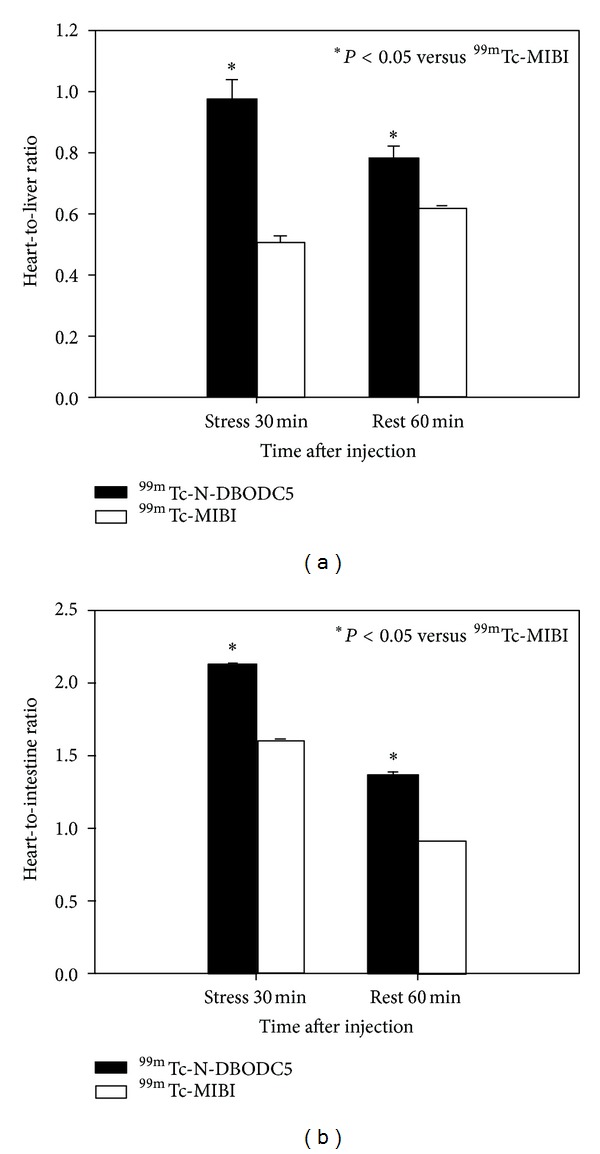
Heart-to-liver ratio and heart-to-intestine ratio measured with an anterior projection images at stress for 30 min and rest for 60 min for ^99m^Tc-N-DBODC5 and ^99m^Tc-MIBI in 46 patients.

Figure 3 depicts the heart-to-liver count ratios (a) and heart-to-intestine count ratios (b) of ^99m^Tc-N-DBODC5 and ^99m^Tc-MIBI in 46 patients. The myocardium-to-liver ratios of ^99m^Tc-N-DBODC5 and ^99m^Tc-MIBI studies were 0.97 ± 0.07 and 0.51 ± 0.02, respectively, at stress. The myocardium-to-liver ratios of ^99m^Tc-N-DBODC5 and ^99m^Tc-MIBI MPI were 0.78 ± 0.04 and 0.62 ± 0.01, respectively, at rest. On the other hand, the mean heart-to-intestine ratios of ^99m^Tc-N-DBODC5 were 2.12 ± 0.01 and 1.36 ± 0.02 for stress and rest images, respectively. The average heart-to-intestine ratios of ^99m^Tc-MIBI were 1.62 ± 0.01 and 0.91 ± 0.00 for stress and rest images, respectively. ^99m^Tc-N-DBODC5 studies had a significantly higher heart-to-organ count ratio compared with ^99m^Tc-MIBI studies (*P* < 0.05). 

### 3.3. Image Quality

In the stress perfusion imaging, ^99m^Tc-N-DBODC5 MPI was superior in quality to ^99m^Tc-MIBI MPI in 37 of the 46 patients studied. In the remaining nine patients, the MPI was judged to be of equal quality. In the resting perfusion imaging, ^99m^Tc-N-DBODC5 images were superior in all the patients studied. In general, the ^99m^Tc-N-DBODC5 MPI had more counts with less statistical noise, less tracer activity below the diaphragm, and desirable heart-to-organ count ratio compared with ^99m^Tc-MIBI MPI.

### 3.4. Gated SPECT Findings

Table 2 shows the results of scan segments between the ^99m^Tc-N-DBODC5 and ^99m^Tc-MIBI MPI. Of the 782 segments that could be interpreted by both techniques, 302 were concordantly normal, 119 were concordantly reversible, and 136 were concordantly nonreversible. ^99m^Tc-N-DBODC5 SPECT detected more reversible, defects than did ^99m^Tc-MIBI SPECT (240 versus 140, *P* < 0.05, McNemar test). ^99m^Tc-MIBI SPECT identified more nonreversible defects than did ^99m^Tc-N-DBODC5 SPECT (312 versus 152, *P* < 0.001, McNemar test). Seventy-one segments interpreted as normal on ^99m^Tc-N-DBODC5 corresponded to nonreversible defects on ^99m^Tc-MIBI SPECT images. In contrast, 12 normal ^99m^Tc-MIBI segments corresponded to nonreversible defects on ^99m^Tc-N-DBODC5 images. When the patterns of uptake (normal, reversible defect, and nonreversible defect) of ^99m^Tc-N-DBODC5 and ^99m^Tc-MIBI were compared, there was concordance in 71% (557/782) of segments.

Table 3 shows the results of left ventricular function parameters in the two techniques used. In this study, the end-diastolic volume (EDV), end-systolic volume (ESV), left ventricular ejection fraction (LVEF), and transient ventricular dysfunction (TID) were assessed by MPI using two tracers. No statistically significant differences in MPI parameters were observed between the ^99m^Tc-MIBI and ^99m^Tc-N-DBODC5 studies. However, the scores of myocardial perfusion defects for the detection of CAD were higher with ^99m^Tc-MIBI than with ^99m^Tc-N-DBODC5 (*P* = 0.012, for SSS; *P* = 0.020, for SDS, resp.).

### 3.5. Detection of CAD

**Figure 4:**
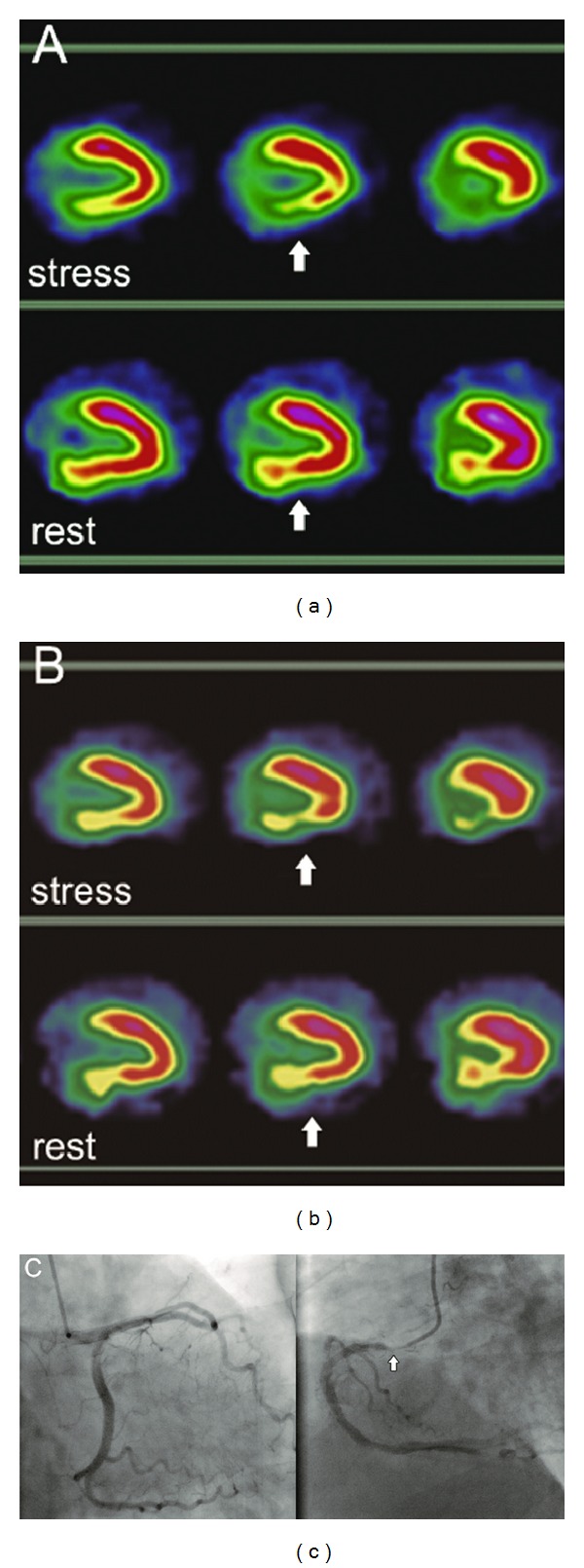
Abnormal MPI in the vertical long-axis slices of a representative patient. Both ^99m^Tc-N-DBODC5 (a) and ^99m^Tc-MIBI (b) images demonstrate an inferoposterior defect (white arrows). The defect is well visualized on two tracer images corresponding to the CA result. This coronary angiography (c) detected a stenosis of 90% in the RCA. The concordance for diagnosis of myocardial ischemia was seen on ^99m^Tc-N-DBODC5 and ^99m^Tc-MIBI studies.

Table 4 shows the sensitivity and specificity of two tracers in diagnosing CAD. Based on CA, overall figures for sensitivity and specificity in identification of CAD were 86% (25/29) and 65% (11/17) for ^99m^Tc-MIBI imaging. On the other hand, the overall sensitivity and specificity for the detection of CAD were 86% (25/29) and 88% (15/17) for ^99m^Tc-N-DBODC5 SPECT studies. The concordance of ischemia diagnosis sensitivity of a representative patient for the two tracers is shown in Figure 4. The accuracy was 0.78 for ^99m^Tc-MIBI and 0.87 for ^99m^Tc-N-DBODC5. Angiography agreement was very good for ^99m^Tc-N-DBODC5 (*κ* = 0.73) and moderate for ^99m^Tc-MIBI (*κ* = 0.52). Specificity and accuracy were not significantly different but better for the ^99m^Tc-N-DBODC5 group when compared to ^99m^Tc-MIBI SPECT (specificity, 88-65%; accuracy, 87-78%).

### 3.6. Detection of Disease in Individual Coronary Vessels

Table 4 also shows the sensitivity and specificity of the two tracers in detecting individual-stenosed vessels. Of a total of 138 arteries in 46 patients, 48 arteries had significant stenoses, and 90 had insignificant lesions or were normal. Overall figures for sensitivity and specificity in identifying individual-stenosed vessels were 67% (32/48) and 86% (77/90) for ^99m^Tc-MIBI imaging; overall sensitivity and specificity to diagnose individual significantly stenosed vessels were 69% (33/48) and 92% (83/90) for ^99m^Tc-N-DBODC5 SPECT studies.

On the other hand, sensitivity and specificity for RCA stenosis vessel lesion detection using ^99m^Tc-MIBI MPI were 87% and 68% compared to 87% and 87% for ^99m^Tc-N-DBODC5 MPI, LAD stenosis (53% sensitivity and 96% specificity for ^99m^Tc-MIBI, 63% sensitivity and 96% specificity for ^99m^Tc-N-DBODC5, resp.), and left circumflex (LCX) (64% sensitivity and 94% specificity for ^99m^Tc-MIBI, 57% sensitivity and 94% specificity for ^99m^Tc-N-DBODC5, resp.). Angiography agreement was very good for ^99m^Tc-N-DBODC5 (*κ* = 0.63 for LAD; *κ* = 0.71 for RCA, resp.) and moderate for ^99m^Tc-MIBI (*κ* = 0.52 for LAD; *κ* = 0.48 for RCA, resp.). In contrast, the angiography agreement was very good for ^99m^Tc-MIBI (*κ* = 0.62 for LCX) and moderate for ^99m^Tc-N-DBODC5 (*κ* = 0.55 for LCX) (Table 4). Overall, the specificity of ^99m^Tc-N-DBODC5 SPECT to detect individual RCA stenosis was better (27/31, 87%) than that of ^99m^Tc-MIBI SPECT (21/31, 68%), despite having no statistical significance.

## 4. Discussion

These preliminary results demonstrate that stress-rest myocardial perfusion SPECT with ^99m^Tc-N-DBODC5 is a sensitive method for detecting CAD and identifying stenosed coronary arteries. For the detection of CAD, more importantly, ^99m^Tc-N-DBODC5 MPI reached good agreement compared with CA (*κ* = 0.73).

### 4.1. Advantages of ^99m^Tc-N-DBODC5

^99m^Tc-N-DBODC5 is a new lipophilic, monocationic, and nitride ^99m^Tc-labeled tracer that is rapidly cleared from the liver after intravenous injection. It possesses good stability under physiological conditions. Importantly, ^99m^Tc-N-DBODC5 exhibits more rapid liver washout than either ^99m^Tc-MIBI or ^99m^Tc-tetrofosmin. For example, at 60 min after injection in rats, the heart/liver ratio of ^99m^Tc-N-DBODC5 is approximately ten times higher than that of ^99m^Tc-MIBI or ^99m^Tc-tetrofosmin. Preclinical studies have shown that ^99m^Tc-N-DBODC5 SPECT can identify previous ischemia as areas of reduced tracer uptake [25, 26]. Furthermore, the rapid liver clearance and high uptake in the myocardium of ^99m^Tc-N-DBODC5 will allow SPECT images of the left ventricle to be acquired early and with excellent quality [22]. The ratios of heart-to-liver were consistent with the previous report study [22]. These characteristics are suitable, particularly for patients with suspected acute coronary syndromes and without diagnostic electrocardiogram, because early diagnosis is needed in such patients for timely therapeutic decision making. ^99m^Tc-MIBI, on the other hand, may require a wait time of up to 1 h for imaging.

In the present study, the heart-to-organ count ratio of ^99m^Tc-N-DBODC5 MPI was superior to that of ^99m^Tc-MIBI in 46 patients. Compared with ^99m^Tc-MIBI MPI, ^99m^Tc-N-DBODC5 MPI had better image quality in most patients. Furthermore, in this study, exercise stress myocardial images were performed 30 min after ^99m^Tc-N-DBODC5 injection, and excellent MPI with high contrast was possible.

### 4.2. Clinical Value of ^99m^Tc-N-DBODC5 for the Detection of CAD

This clinical trial assesses the diagnostic value of this agent to detect CAD comparing it with stress ^99m^Tc-MIBI MPI and CA.

In this study, compared with ^99m^Tc-MIBI MPI, ^99m^Tc-N-DBODC5 MPI indicates the same sensitivity and better specificity and accuracy to detect coronary disease in patients, although none of the differences were significant. On the other hand, more importantly, angiography agreement was very good for ^99m^Tc-N-DBODC5 (*κ* = 0.73) and moderate for ^99m^Tc-MIBI (*κ* = 0.52); thus, compared with ^99m^Tc-MIBI MPI, it provides a high degree of concordance for the evaluation of CAD, specifically in excluding perfusion abnormalities in patients with suspected CAD. Possible reasons for this difference may be fast liver clearance, favorable heart-to-organ ratio, and high image quality. ^99m^Tc-N-DBODC5 with rapid liver clearance may significantly reduce the photon scatter from the liver into the inferoposterior walls. This reduces the artifactual decreased myocardial perfusion and improves the diagnostic accuracy for the detection of CAD compared with other ^99m^Tc-labeled perfusion agents [24].

Unsurprisingly, Braat et al. [35] and Germano et al. [36] noted that technetium-labeled MPI agents with a fast liver clearance can significantly reduce the photon scatter from the liver into the inferior walls, but the radioactivity may be transferred to the gastrointestinal area, and increased bowel and gastric activity were seen as a problem associated with high liver uptake in the visual and quantitative interpretation of the inferoposterior myocardial walls. In this study, bowel uptake was frequently seen on the ^99m^Tc-N-DBODC5 images, but did not result in any nondiagnostic scans.

For the detection of coronary disease in patients, ^99m^Tc-MIBI produced abnormal results for MPI in four patients who had no angiographically detected stenosis. Some of these false-positive results may be due, in part, to the liver-to-heart artifacts. In this study, we could classify fixed perfusion defects as soft-tissue attenuation artifacts or infarcts by using gated SPECT. Because an artifactual defect would show normal contraction (wall motion or thickening) on a gated image, artifacts can be differentiated from a true infarct [37].

**Figure 5:**
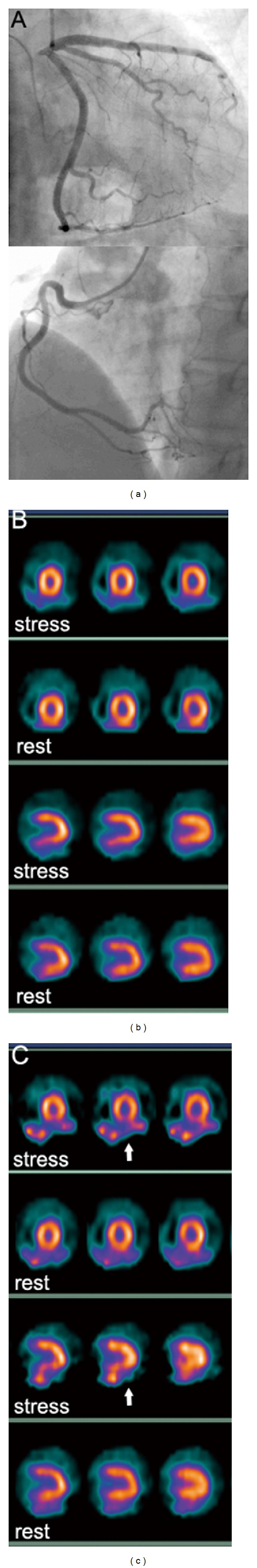
Serial short-axis and vertical long-axis slices of stress-rest ^99m^Tc-N-DBODC5 images (b) and stress-rest ^99m^Tc-MIBI images (c) of a representative patient with normal CA (a). Because of intense uptake of technetium ^99m^Tc-MIBI in the liver, high liver background activity can be observed. Furthermore, a false-positive myocardial perfusion defect was also seen in the inferoposterior wall segments supplied by the RCA territory (white arrows). Importantly, however, at stress and rest, the inferoposterior wall segments of ^99m^Tc-N-DBODC5 images are clearly separated from the subdiaphragmatic activity.

For the detection of individual vessel stenosis, on the other hand, angiography agreement was very good for ^99m^Tc-N-DBODC5 (*κ* = 0.71 for RCA) and moderate for ^99m^Tc-MIBI (*κ* = 0.48 for RCA). For LAD and LCX arteries stenosis, the diagnosis of myocardial ischemia on ^99m^Tc-N-DBODC5 and ^99m^Tc-MIBI study had the same specificity; and it was similar to that of two tracer to diagnose sensitivity and accuracy of myocardial ischemia. Abnormal results of myocardial images in individual RCA vessel stenosis (six arteries-stenosed vessels for RCA) were obtained in the ^99m^Tc-MIBI studies, while CA and ^99m^Tc-N-DBODC5 studies showed normal findings in the same individual RCA vessel territories. Some of these false-positive results may be due to photon scatter from the liver and intestine into the inferoposterior walls. ^99m^Tc-MIBI concentration located below the left diaphragm (i.e. liver and bowel) may cause an artifactual perfusion defect in the adjacent myocardial, a phenomenon known as the “liver-heart artifact” [36, 38]. Moreover, the inferior and inferoposterior regions were dominated mainly by RCA territories corresponding to the myocardial short axis and vertical long axis; while arteries regions which LAD and LCX dominated were less interfered from “liver-heart artifact.” Finally, for clinical applications, ^99m^Tc-N-DBODC5 offers better determination of detects, particularly in the inferoposterior wall on myocardial images. A typical example of a false-positive perfusion defect in the inferior wall is shown in Figure 5.

On a segment-to-segment basis, complete agreement between the two imaging agents occurred in 71% of segments. However, more myocardial segments were nonreversible on ^99m^Tc-MIBI images than on ^99m^Tc-N-DBODC5 images. On the contrary, ^99m^Tc-N-DBODC5 MPI had a higher reversible defect than that of ^99m^Tc-MIBI. A possible reason for this difference may be due, in part, to the “liver-heart artifact.” The presence of high liver activity adjacent to the inferior wall results from oversubtraction of activity from the inferior wall. Therefore, the more “liver-heart artifact” in the inferoposterior wall on myocardial images, the more false nonreversible defects in the inferoposterior wall on myocardial perfusion segmental analysis. In other words, the less reversible defects in the inferoposterior wall were deduced on ^99m^Tc-MIBI MPI segmental analysis.

More importantly, however, the main advantage and clinical value of ^99m^Tc-N-DBODC5 can improve diagnostic specificity and reduce the false-positive diagnosis of patients and associated treatment fees. 

## 5. Limitations

In this study, we did not attempt to assess the absolute diagnostic accuracy of ^99m^Tc-N-DBODC5, because definitive conclusions in this regard can only be drawn by studying larger patient cohort. In addition, although ^99m^Tc-N-DBODC5 did not track flow as well as ^201^Tl, the magnitudes of the pharmacologic stress-induced perfusion defects were comparable to those previously reported for ^99m^Tc-tetrofosmin. Thus, direct comparison between ^99m^Tc-N-DBODC5 and ^99m^Tc-tetrofosmin should be further proved in the same patient population. Overall, ^99m^Tc-N-DBODC5 MPI will become an important diagnostic tool in the evaluation of myocardial perfusion.

## 6. Conclusion

This preliminary clinical study showed that ^99m^Tc-N-DBODC5 and ^99m^Tc-MIBI MPI provide comparable diagnostic information for patients undergoing exercise rest for detection of CAD. In addition,   ^99m^Tc-N-DBODC5 does not exhibit the disadvantages of ^99m^Tc-MIBI in this study. By contrast, because of its high heart-organ count ratio in comparison to ^99m^Tc-MIBI, it improves high degree of diagnostic concordance in defining or excluding perfusion abnormalities in patients with CAD. Therefore, it can become the agent of choice for the evaluation of myocardial perfusion and ventricular function in patients with CAD.

## Figures and Tables

**Table 1:** Study group.

Parameter	Value
*n*	46
M/F	31/15
Age (yr)	60.08 ± 8.58 (39–74)
Hypertension	31
Hyperlipidemia	19
Diabetes mellitus	14
Smoking	26
Trigger of chest pain	
Effort	22
Rest	13
Not specific	11
ECG abnormality	11
ST-T elevation	3
ST-T depression	8
≥50% of luminal narrowing	29
One-vessel disease	14
Two-vessel disease	11
Three-vessel disease	4

Data are presented as mean ± SD or number (%) as appropriate.

**Table 2:** The comparison of myocardial perfusion in a total of 782 segments with ^99m^Tc-N-DBODC5 and ^99m^Tc-MIBI SPECT exercise imaging.

^ 99m^Tc-N-DBODC5	^ 99m^Tc-MIBI			Total
	Normal	Reversible defect	Nonreversible defect*	
Normal	302 (39%)	17 (2%)	71 (9%)	390
Reversible defect*	16 (2%)	119 (15%)	105 (13%)	240
Nonreversible defect	12 (2%)	4 (1%)	136 (17%)	152

Total	330	140	312	782

Data are presented as number; *P < 0.001,^ 99m^Tc-MIBI versus ^99m^Tc-N-DBODC5 for nonreversible defect segments; *P < 0.05, ^99m^Tc-N-DBODC5 versus ^99m^Tc-MIBI for reversible defect segments; McNemar test was used.

**Table 3:** MPI findings of two tracers on left ventricular function parameters and ischemia scores in 46 patients.

Parameter	^ 99m^Tc-MIBI	^ 99m^Tc-N-DBODC5
LVEF (%)		
Exercise	54.2 ± 11.3	56.7 ± 9.2
Rest	63.1 ± 8.5	64.8 ± 7.9
EDV (mL)		
Exercise	81.5 ± 18.6	83.2 ± 16.3
Rest	97.3 ± 16.4	99.1 ± 13.7
ESV (mL)		
Exercise	43.2 ± 9.7	41.4 ± 12.3
Rest	59.4 ± 13.6	60.2 ± 9.8
SSS	12.1 ± 1.4^★^	9.6 ± 1.6
SRS	7.2 ± 0.8	7.9 ± 0.9
SDS	4.2 ± 0.5^★^	3.6 ± 0.4
TID	0.96 ± 0.06	0.94 ± 0.02

Data are presented as mean ± SD or number (%) as appropriate; ^★^statistically significant ^99m^Tc-MIBI versus ^99m^Tc-N-DBODC5 (*P* < 0.05); paired Student's *t*-test was used; LVEF: left ventricular ejection fraction; EDV: end-diastolic volume; ESV: end-systolic volume; SSS: summed stress scores; SRS: summed rest scores; SDS: summed difference scores; TID: transient ventricular dysfunction.

**Table 4:** Sensitivity, specificity, and diagnostic accuracy of scintigraphic perfusion studies and agreement with coronary angiography.

	Overall		LAD		LCX		RCA	
	MIBI	DBODC	MIBI	DBODC	MIBI	DBODC	MIBI	DBODC
No. of disease	25	25	10	12	9	8	13	13
Sensitivity (%)	86	86	53	63	64	57	87	87
Specificity (%)	65	88	96	96	94	94	68	87
Accuracy (%)	78	87	78	83	85	83	74	87
^▲^Kappa (*κ*)	0.53	0.73	0.52	0.63	0.62	0.55	0.48	0.71

Data are presented as number (%); MIBI = ^99m^Tc-MIBI, DBODC = ^99m^Tc-N-DBODC5; LAD: left anterior descending coronary artery; LCX: left circumflex coronary artery; RCA: right coronary artery. ^▲^CA was used as the “gold standard” for the calculation of the *κ*, which is determine between SPECT MPI and CA. If there is no agreement, *κ* = 0.20–0.39; moderate agreement, *κ* = 0.40–0.59; very good agreement, *κ* = 0.60–0.79; excellent agreement, *κ* = 0.80–1.00.

## Abbreviations

CAD: Coronary artery disease
MPI: Myocardial perfusion imaging
CA: Coronary angiography
SPECT: Single-photon emission computed tomography
PET: Positron emission computed tomography
^99m^Tc-MIBI: Technetium 99m sestamibi
^99m^Tc-N-DBODC5: ^99m^Tc(N)(DBODC)(PNP5)
^201^Tl: Thallium-201
^99m^Tc-tetrofosmin: Technetium-99m-tetrofosmin
ROI: Regions of interest
LAD: Left anterior descending artery
LCX: Left circumflex artery
RCA: Right coronary artery
SSS: Summed stress score
SRS: Summed rest score
SDS: Summed difference score
EDV: End-diastolic volume
ESV: End-systolic volume
LVEF: Left ventricular ejection fraction.
